# Magnetic Resonance Spectroscopy: An In Vivo Molecular Imaging Biomarker for Parkinson's Disease?

**DOI:** 10.1155/2014/519816

**Published:** 2014-09-11

**Authors:** Rosella Ciurleo, Giuseppe Di Lorenzo, Placido Bramanti, Silvia Marino

**Affiliations:** ^1^IRCCS Centro Neurolesi “Bonino-Pulejo,” S.S. 113 Via Palermo, Contrada Casazza, 98124 Messina, Italy; ^2^Department of Biomedical Sciences and Morphological and Functional Imaging, University of Messina, 98124 Messina, Italy

## Abstract

Parkinson's disease (PD) is a neurodegenerative disorder caused by selective loss of dopaminergic neurons in the substantia nigra pars compacta which leads to dysfunction of cerebral pathways critical for the control of movements. The diagnosis of PD is based on motor symptoms, such as bradykinesia, akinesia, muscular rigidity, postural instability, and resting tremor, which are evident only after the degeneration of a significant number of dopaminergic neurons. Currently, a marker for early diagnosis of PD is still not available. Consequently, also the development of disease-modifying therapies is a challenge. Magnetic resonance spectroscopy is a quantitative imaging technique that allows in vivo measurement of certain neurometabolites and may produce biomarkers that reflect metabolic dysfunctions and irreversible neuronal damage. This review summarizes the abnormalities of cerebral metabolites found in MRS studies performed in patients with PD and other forms of parkinsonism. In addition, we discuss the potential role of MRS as in vivo molecular imaging biomarker for early diagnosis of PD and for monitoring the efficacy of therapeutic interventions.

## 1. Introduction

Parkinson's disease (PD) is the most common neurodegenerative movement disorder. The disease affects approximately 7 million people globally and has a very high socioeconomic impact. Clinically, PD is characterized by bradykinesia, akinesia, muscular rigidity, postural instability, and resting tremor, including also nonmotor symptoms such as cognitive and psychiatric impairment [1]. Neuropathological hallmarks of PD are degeneration of dopaminergic neurons in substantia nigra pars compacta (SNc) and formation of inclusions called Lewy bodies, mainly composed of α-synuclein, within the central and peripheral nervous system [2, 3]. The loss of dopaminergic neurons in the SNc results in decreased levels of dopamine in the putamen of the dorsolateral striatum, leading to dysfunction of direct and indirect pathways of movement control that involve corticobasal ganglia-thalamocortical loops [2].

In PD, the patients fulfill the clinical criteria when approximately 60–70% of nigrostriatal neurons are degenerated and 80% of content of striatal dopamine is reduced [4]. In the “preclinical” PD phase nonmotor symptoms, such as olfactory dysfunction, constipation, rapid eye movement behavior disorder, mood disorders, and depression, precede motor symptoms reflecting the dysfunction of dopaminergic or nondopaminergic neurons. This clinical condition describes a stage of disease called “prodromal” [5]. Detection of prodromal PD phase is becoming an important goal for determining a definite diagnosis and for choosing a suitable treatment strategy. Currently, PD treatment is symptomatic. One of the major challenges in PD is the development of disease-modifying therapies, such as neuroprotective or cell-base restorative agents. The clinical symptoms appear after the degeneration of a significant number of dopaminergic neurons and, in this advanced stage, the disease-modifying therapies may be ineffective to attenuate progression of the neurodegeneration. Thus, the identification of specific and sensitive biomarkers is extremely important to facilitate early and differential diagnosis, monitor disease progression, and assess efficacy of current and future treatments.

Magnetic resonance spectroscopy (MRS) is a noninvasive imaging technique, for exploration in vivo of intracellular metabolic status, and may provide a neuroimaging biomarker of normal biological and pathological processes or response to a therapeutic intervention. This paper presents an overview of MRS and its implications for detection in vivo of neurodegeneration processes in PD. In addition, the potential role of MRS as in vivo molecular imaging biomarker to confirm early and differential PD diagnosis and to assess response to therapy is discussed.

We performed a review of peer-reviewed literature using Pubmed/Medline. The search was limited to studies reported in English language and published from January 1990 to February 2014 (Tables 1, 2, and 3). A combined search was performed using the following terms: magnetic resonance spectroscopy, proton magnetic resonance spectroscopy, phosphorus magnetic resonance spectroscopy, Parkinson's disease, atypical parkinsonian disorders, progressive supranuclear palsy, multiple-system atrophy, and corticobasal degeneration, N-acetylaspartate, differential diagnosis, and dopaminergic therapy. We selected the articles according to the following criteria: (1) MRS studies that involved humans; (2) studies that compared spectra of PD patients to those of healthy controls; and (3) single and multivoxel MRS studies.

## 2. Proton Magnetic Resonance Spectroscopy

MRS is a noninvasive imaging technique that can be used to measure the concentrations of different low-molecular weight chemicals. The technique is based on the same physical principles of magnetic resonance imaging (MRI), that is, the detection of energy exchanges between external magnetic fields and specific nuclei within atoms. MRS is used in vivo for the study of different nuclei, including ^1^H, ^31^P, ^13^C, ^15^N, ^19^F, and ^23^Na. The main nucleus studied today in neurospectroscopy is ^1^H, which provides information on markers of neurons, myelin, energy metabolism, and other metabolically active compounds.

The metabolites detectable with proton MRS (^1^HMRS) include the prominent resonances of N-acetylaspartate (NAA), choline-containing compounds (Cho), creatine + phosphocreatine (Cr), myoinositol (mI), lactate (Lac), and a variety of other resonances that might not be evident depending on type and quality of spectra as well as on the pathological condition [6].

NAA, which resonates at 2.02 parts per million (ppm), represents the largest proton metabolic concentration in the human brain after water. Indeed, the concentration of NAA reaches the order of 10 *µ*mol/g. NAA is widely interpreted as a neuronal marker and implicated in several neuronal processes, including lipid and protein synthesis, mitochondrial functioning, and osmoregulation. NAA synthesis occurs in mitochondria and requires acetyl-CoA and L-aspartic acid as substrates. NAA has been proposed to serve as a mitochondrial shuttle of acetyl-CoA used for fatty acid synthesis. NAA is reduced in many brain disorders in presence of neuronal or axonal loss. The Cho peak (3.2 ppm) represents a combination of several choline-containing compounds, including free Cho, phosphorylcholine, and glycerophosphorylcholine, and to a small extent acetylcholine. Free Cho acts as a precursor to acetylcholine, while glycerophosphorylcholine is a product of breakdown of membrane phosphatidylcholine and acts as an osmoregulator. The concentration of Cho is relatively low on the order of 0.5 to 1.5 *µ*mol/g. The Cho peak is often viewed as a marker of membrane turnover or inflammation in ^1^HMRS studies. The Cr peak (3.03 ppm) is composed of creatine and phosphocreatine. These metabolites buffer the energy use and energy storage of cells. The concentration of total Cr is estimated on the order of 8 to 9 *µ*mol/g. Cr concentration is often used as an internal standard because it is considered to be relatively stable, showing slow increase with age. Thus, total Cr is often used as an internal reference (i.e., a denominator in metabolite signal ratio). The mI peak (3.56 ppm) represents the presence of myoinositol and myoinositol phosphate. MI is suggested as a glial marker, osmoregulator, intracellular messenger, and detoxifying agent. The Lac (1.3 ppm) is an end product of anaerobic glycolysis; thus the increase in Lac concentrations often serves as an index of altered oxidative metabolism, that is, in ischemia, hypoxia, and cancer [6]. The amino acids glutamine (Glu), glutamate (Gln), and *γ*-aminobutyric acid (GABA) (2.1–2.4 ppm) are involved in excitatory and inhibitory neurotransmission. MRS at high field strengths improves the quantitation of these compounds [7]. MRS is implemented as single-voxel and multivoxel method. Single-voxel spectroscopy detects the signal from a single region during one measurement, whereas multivoxel or MR spectroscopic imaging (MRSI) or chemical shift imaging (CSI), using additional phase-encoding pulses, obtains the signal from multiple regions at the same time and provides the information of spatial distribution of major cerebral metabolites [8]. The metabolite concentrations are expressed in terms of semiquantitative ratios such as NAA/Cr, NAA/Cho, Cho/Cr, and mI/Cr. In relative quantification, one of the metabolite peaks measured is used as the concentration standard and serves as the denominator of peak ratios. As a result, the total number of quantifiable metabolites is decreased by one. Furthermore, alterations in the peak ratio do not necessarily reflect a change in the concentration of the numerator. The alteration may be caused by change in the concentration of the numerator, the denominator, or both or may be due to changes in relaxation behavior. The assumption that the concentration of certain reference metabolites (e.g., total creatine and choline) remains constant may be incorrect under normal conditions, as well as in many pathologic states. It is therefore advisable to obtain concentration expressed in standard units (such as millimoles per kilogram wet weight) by applying absolute quantification.

MRS is applied to help researchers in the understanding of pathophysiological mechanisms and clinicians in the diagnosis and follow up of neurological disorders. Currently, in care of PD patients, MRS coupled with a careful clinical assessment is assuming a certain importance for the differential diagnosis of PD with initial motor symptom from atypical parkinsonian disorders (APDs), supporting the diagnostic accuracy of the neurologist's assessment, and, consequently, improving the disease management.

## 3. Metabolic Changes Detected by ^**1**^HMRS in Parkinson's Disease

The first ^1^HMRS studies were designed to identify possible alterations of metabolic status of cortical-basal ganglia structures involved in motor dysfunctions in PD patients versus healthy control subjects (Table 1). Abnormal ^1^HMRS spectra were reported in basal ganglia. In particular, a significant reduction of NAA/Cho ratios was found in the lentiform nucleus of PD patients compared with control subjects [9]. Choe et al. [10] showed asymmetric decrease of NAA/Cr ratios in the contralateral SN to the symptomatic side in PD with unilateral symptoms. However, a study reported a significant increase of total Cr levels in prefrontal cortex, but no change in NAA and Cho in SN of PD patients [11]. Other studies reported metabolic alterations also in cortical structures. Reduced NAA and Cho levels in temporoparietal cortex [12, 13] and reduced NAA levels in motor cortex [14], posterior cingulated cortex [15], and presupplementary motor area [16] were observed. Some studies reported no metabolite differences between the PD patients and the control subjects in either metabolite ratios or absolute concentration of NAA, Cho, and Cr in cortical-basal ganglia loop [17–20]. A ^1^H-MRSI study of Tedeschi et al. [19] found that there were no significant differences between PD patients and control subjects of NAA, Cho, and Cr ratios in brainstem, caudate, thalamus, lentiform nucleus, and association cortices. These findings were in agreement with previous single-voxel ^1^H-MRS studies that showed no significantly reduced NAA in lentiform nucleus [17] and putamen and thalamus [18].

The recent development of ^1^HMRS at high magnetic field strengths led ^1^HMRS to play a more important role as imaging tool in the identification of metabolite changes in PD. Indeed, ^1^HMRS of the brain with high magnetic field strengths has many advantages which include better signal-noise ratio and increased spectral, spatial, and temporal resolution, allowing detection of a greater number of metabolites (such as Glu/Gln and GABA), more reliable estimation of peak area, and hence more precise quantification compared with ^1^HMRS at 1.5 Tesla [26].

A 3D ^1^HMRSI at 3 Tesla study reported in PD patients changes not only of NAA/Cr ratios but also of mI/Cr ratios, with significant differences of two metabolites between rostral and caudal SN regions [21]. In particular, in the rostral SN regions PD patients showed a trend towards decreased NAA/Cr and mI/Cr ratios compared with control subjects whereas in the caudal SN regions the metabolite ratios were increased in PD patients compared with control subjects. Another 3 Tesla ^1^HMRSI study investigated metabolite distribution throughout the whole brain. In particular, reduced NAA/Cr and Cho/Cr ratios in bilateral temporal gray matter and increased Cr in right temporal gray matter in PD patients versus control subjects were found [22].

^1^HMRS at high magnetic field strengths was used also to detect and quantify Glu and GABA, which did not show abnormalities in ^1^HMRS at 1.5 Tesla studies [9, 20]. ^1^HMRS at 3 Tesla studies found reduced Glu levels in the posterior cingulated gyrus [23], but not in the lentiform nucleus [27] in PD patients compared with control subjects. The cortical Glu reduction is confirmed by a study on animal model of PD. In this study, performed on 1-methyl-4-phenyl-1,2,3,6-tetrahydropyridine (MPTP)-lesioned nonhuman primates, Fan et al. [28] found that Glu levels were reduced in the primary motor cortex on the side ipsilateral to the lesion. A ^1^HMRS at 4 Tesla study, comparing the metabolic profile of 10 PD patients with that of matched controls, reported decreased Glu, NAA, and glutathione levels and increased Cho levels in SN of PD patients [24]. In addition, the authors reported a fourfold higher GABA/Glu ratio in SN versus cerebral cortex. A ^1^HMRS at 7 Tesla study reported that GABA concentrations in the pons and putamen were significantly higher in mild to moderate PD patients than healthy controls [25]. These findings on GABA are consistent with animal model studies that found elevated GABA levels in the striatum [29–31].

Significant alterations in neurochemical levels may provide new evidences able to elucidate the pathophysiological mechanisms underlying of PD. Reduced NAA levels, observed in all cerebral structures of patients with PD by ^1^HMRS, reflect not only wide neuronal degenerations, which involves the corticobasal ganglia-thalamocortical networks, but also metabolic dysfunctions. Indeed, since NAA is synthesized in neuronal mitochondria in an energy-dependent manner, its decrease in PD would confirm the hypothesis that the dysfunction of the mitochondrial electron transport chain, as a result of a defect in complex I, is a primary or secondary event in PD pathogenesis [32]. Although the reduction of NAA levels is a condition that may occur also in other neurodegenerative diseases, this finding in PD patients may be indicative of impairments in mitochondrial metabolic system that hypothetically contribute to neuronal degeneration. The mitochondrial dysfunction in PD is also supported by a study of Bowen et al. [33], who reported in occipital lobe of PD patients high levels of Lac, metabolite which accumulates in most disease associated with a deficiency in mitochondrial oxidative metabolism.

^1^HMRS of the NAA levels might represent a useful in vivo imaging biomarker also for the prediction of cognitive decline in PD. Interestingly, decreased NAA/Cr ratios were found in anterior cingulated cortex of PD patients compared with controls. These low NAA levels significantly correlated with poorer executive function and more severe psychotic symptoms in PD patients [34]. In addition, changes of NAA and Cho levels in early cognitive impairment phase of PD patients were observed. In particular, a recent ^1^HMRS at 3 Tesla reported that NAA/Cr ratios in the occipital lobe of PD patients with mild cognitive impairment were lower than in control subjects and Cho/Cr ratios in the posterior cingulated of PD patients with mild cognitive impairment were higher than in healthy control subjects and cognitively normal PD patients [35].

The cerebral regional variability of Cho levels observed in some studies [12, 22, 24, 35] makes it difficult to understand what role the Cho can have in the pathophysiology of PD. The reduction of Cho levels observed in some studies [12, 22] might be related to damage of membrane structure of neuronal cells within the corticostriatal system. On the other hand, the trend to increase of Cho and Cr [11, 22, 35] could be a minimal sign for neuroinflammation. Indeed, since higher concentrations of Cho and Cr are present in glial cells than neurons, they may be elevated in neuroinflammation condition [36], although an increase of glial marker mI has been not reported in PD. Therefore, the available evidence is not sufficient to ascribe to Cho and Cr a role as biomarkers of neuroinflammation in PD. Finally, ^1^HMRS changes in Glu and GABA levels reported in PD may reflect alterations of the balance between excitatory and inhibitory processes in the corticobasal ganglia-thalamocortical networks involved in motor control.

## 4. ^**1**^HMRS in Parkinson's Disease and Atypical Parkinsonian Disorders

In early stage of PD the motor symptoms can easily be mistaken for any number of disorders. Indeed, it is very likely that the PD may be confused with various APDs, such as progressive supranuclear palsy (PSP), multiple-system atrophy (MSA), especially the Parkinson's variant of multiple-system atrophy (MSA-P), and corticobasal degeneration (CBD). A differentiation of these clinical entities may be challenging, particularly in the early stages of motor symptoms of the disease, where overlapping clinical signs lead to a high rate of misclassification. However, a differentiation between APDs and PD is important for making easier early diagnosis and for choosing a specific treatment strategy.

MRS plays an important role in the differentiation of PD from APDs, especially in early stage of disease where a differentiation of these conditions is not easy (Table 2).

^1^HMRS of striatal structures might differentiate PD from APDs by virtue of reduced NAA/Cr ratios in MSA but not PD. In particular, ^1^HMRS showed reduced NAA/Cr ratios in the lentiform nucleus in six of seven MSA-P cases, whereas normal levels of putaminal NAA were found in eight of nine PD subjects [17]. A study by Abe et al. [37] showed that, as compared to normal controls, patients with PSP, CBD, MSA, and PD had significant reduction of NAA/Cr ratios in the putamen, whereas patients with PSP, CBD, and MSA, but not PD, had significant reduction of the NAA/Cr ratios also in the frontal cortex.

Moreover, patients with CBD showed significant reduction of NAA/Cr ratios in the frontal cortex and putamen as compared to patients with PD and MSA. Patients with PSP showed a significant reduction of NAA/Cr ratios in the putamen as compared with patients with PD and MSA. Guevara et al. [38], using MRSI on a 1.5 Tesla scanner, found that patients with PSP and MSA-P had lower NAA concentrations in the pallidum, putamen, and lentiform nucleus compared to healthy controls and patients with PD. In another ^1^HMRS study, in which the single voxel was localized to the lentiform nucleus, Federico et al. [39] showed that NAA/Cho ratios were significantly reduced in MSA and in PSP patients compared to PD patients and to controls. Moreover the NAA/Cr ratios were significantly reduced in MSA, PSP, and PD patients compared to controls, but only in MSA compared to PD patients. However, other MRS studies showed reduced NAA/Cr and NAA/Cho ratios in the lentiform nucleus not only in APD but also in PD [42]. A study investigated 24 patients with MSA compared to 11 PD patients and 18 controls by applying multiple regional single voxel ^1^HMRS including putamen, pontine basis, and cerebral white matter (WM) at 3 Tesla [40]. Significant NAA/Cr reductions have been shown in the pontine basis of patients with both MSA-C (cerebellar ataxia variant of MSA) and MSA-P, while putaminal NAA/Cr was only reduced in the patients with MSA-P. Eight of the 11 MSA-P patients compared to none of the PD and control group were classified correctly by combining individual NAA/Cr reductions in the pontine basis and in the putamen. These results suggest that combined assessment of NAA/Cr in the pontine basis and putamen by higher magnetic field may be effective in differentiating MSA-P from PD in terms of the high specificity of reduced NAA/Cr in the pontine basis or in the putamen in patients with MSA-P. A recent 3D ^1^H-MRSI study at 3 Tesla demonstrated a clear differentiation between PD and APDs, by comparing NAA/Cr ratios in SN regions [41]. In particular, for PD patients, NAA/Cr ratios in the caudal voxels were greater than those in the rostral voxels, whereas for healthy controls and APDs patients these ratios were reversed.

Overall, the striatal NAA differences found in APDs but not in PD patients compared with healthy controls contrast with the findings of the study of Gröger et al [41]. These authors, using ^1^H-MRSI at higher magnetic field strengths, showed NAA differences only in PD patients and attributed these findings to neuronal loss in the SNc as primary pathological mechanism of PD. It is likely that the high-field MRSI, measuring metabolite changes from several areas of the brain simultaneously, provides more reliable and accurate results, making ^1^HMRSI of the NAA, as expression of neuronal integrity and function, an in vivo imaging biomarker for differential diagnosis.

## 5. ^**1**^H- and ^**31**^P-MRSI in Early Diagnosis of Parkinson's Disease

Phosphorus (^31^P)-MRSI is an imaging technique that measures the levels of compounds related to the energy metabolism of the brain including low-energy metabolites, such as free phosphate (Pi) and adenosine mono- and diphosphate (AMP and ADP), and the high-energy phosphates such as adenosine triphosphate (ATP) and phosphocreatine (PCr). Since mitochondrial dysfunction seems to be an early event inducing PD, imaging techniques such as ^31^P-MRSI, able to detect a possible alteration of the brain energy metabolism, could be useful tool for early diagnosis of PD.

Combined ^31^P- and ^1^H-MRSI at 3 Tesla measuring absolute ADP, ATP, Cr, and PCr concentrations in two well-defined cohorts of patients with early and advanced PD have been performed to evaluate brain energy metabolism [43]. In the putamen and midbrain of both PD groups compared to control a bilateral reduction of high-energy phosphates such as ATP and PCr as final acceptors of energy from mitochondrial oxidative phosphorylation was found. In contrast, low-energy metabolites such as ADP and Pi were within normal ranges. Patients with early PD, with clearly lateralized motor symptoms, exhibited a significant reduction of putamen high-energy phosphates in the less affected hemisphere with a less pronounced dopaminergic cell loss. Therefore, mitochondrial dysfunction would seem to be a rather early occurring event in the pathophysiology of dopaminergic degeneration in PD, although a recent ^31^P- and ^1^H-MRSI study at 3 Tesla did not detect metabolic abnormalities in early PD compared with controls [44]. Since few ^31^P-MRSI studies explored brain energy metabolism in PD, it is not still clear if this technique may be an imaging biomarker for detection of mitochondrial dysfunction and then for early diagnosis of PD.

## 6. ^**1**^H-MRSI in Detection of Metabolic Changes in Parkinson's Disease after Treatment

Currently, the available drugs for treating motor symptoms of PD include the replacement therapy with dopamine precursor Levodopa, MAO-B inhibitors, and more recently direct-acting dopamine agonists. When pharmacological treatments are not adequate to control symptoms, surgical techniques such as deep brain stimulation can ameliorate the motor symptoms of PD.

Some ^1^HMRS studies investigated the effects of PD therapy on neurochemical and metabolic profile in cortical-basal ganglia structures (Table 3). In a study evaluating the metabolism of striatum of PD patients versus control subjects, Holshouser et al. [45] reported that NAA/Cho ratios were significantly low in PD patients who did not use levodopa/carbidopa, whereas the ratios were normal in levodopa-treated patients compared with controls. Similarly, Ellis et al. [46] found a significant reduction in putaminal NAA/Cho ratios contralateral to the most affected side in 9 drug-naïve patients with idiopathic PD, but not the 7 levodopa-treated patients compared with controls.

These data suggest that dopaminergic treatment may affect NAA levels in the striatum of PD patients, despite the fact that only prospective studies, in which cerebral metabolic levels are assessed by MRS before and after therapy, could confirm this hypothesis. In a study of Clarke et al. [20] the lentiform nucleus of five PD patients was studied by ^1^HMRS before and 10 minutes after administration of apomorphine. No metabolic differences in NAA, Cho, Cr, and Glu + Gln levels between PD patients and control subjects and between spectra obtained from patients before and after apomorphine therapy were detected. However, a ^1^H-MRS study reported that dopaminergic therapy effected the neurochemical status of the motor cortex in de novo PD patients [47]. Indeed, this study showed an increase in Cho/Cr and in NAA/Cr ratios in the motor cortex and an improvement in motor performance in PD patients 6 months after pergolide treatment. In the same way, a ^1^H-MRS study evaluating the changes in the concentrations of some brain metabolites, in PD patients before and after deep brain stimulation of bilateral subthalamic nucleus, found that after the treatment the cortical NAA/Cho and NAA/Cr ratios were increased significantly and correlated highly with clinical improvement of motor performances [48]. These findings suggest that the clinical treatment-induced improvement might be the result of partial restoration of neuronal functions which in turn may increase the cortical metabolite levels. Overall, NAA recovery could be used as a biomarker of neuronal function for monitoring the response to pharmacological and nonpharmacological therapy of PD.

## 7. Conclusions and Future Directions

PD is a neurodegenerative disease whose insidious onset makes its early diagnosis difficult, which is important to slow down the disease progression and optimise the therapy. During the past two decades, significant progresses have been made in the discovery and assessment of potential biomarkers for the early differential diagnosis and monitoring treatment efficacy.

In vivo MRS can provide a useful and objective tool for detection of cerebral metabolic changes in patients with PD and has been shown to meet many of criteria of an ideal imaging biomarker. Indeed, MRS has good test-retest reliability and, compared with other in vivo imaging biomarkers, such as positron emission tomography (PET) and single photon emission tomography (SPECT), is noninvasive and cheap, and it does not require contrast agents for the molecular imaging involving exposure to radioactive substances. In addition, compared with some in vitro molecular biomarkers, such as mRNA and protein expression levels, that require a complex analysis, MRS is not restricted to specialized centres to perform the analysis, making its extension to general public health centres possible.

The recent technical advances of MRS, including the availability of higher magnetic fields and the development of reliable methods for absolute metabolite quantification and for a better identification of metabolite signals, allowed achieving in vivo detailed information on pathophysiology of PD. In particular, the reduction of NAA levels in cortical-basal ganglia networks reflects neuronal loss and mitochondrial metabolic dysfunction in PD. On the same time, changes of Glu and GABA concentrations detected in vivo in basal ganglia of PD patients could be suggestive of dysfunction of neuronal excitatory and inhibitory activities which are involved in the control of movements. Several studies demonstrated the usefulness of MRS to achieve a differential diagnosis of PD versus other forms of parkinsonism, especially in early stages of disease in which signs and symptoms of different forms of parkinsonism have greater overlap. In addition, there is evidence that MRS may be an in vivo imaging biomarker not only for early and differential diagnosis but also for treatment of PD. However, the studies performed so far are extremely heterogeneous in terms of number of enrolled PD patients, MRS techniques used to identify and to process the metabolite signals, and methods used to calculate the metabolite concentrations. Moreover, technical MRS factors, including different echo- and relaxation times, voxel sizes, field strength, and pulse sequence, may be responsible for result variations observed in some studies. Therefore, multicenter studies on larger samples of PD patients, MRS at high magnetic fields, standardized methods for acquisition and processing of spectroscopic metabolite signals, and use of the absolute quantification of tissue metabolite concentrations are required to definitively ascribe to MRS the role of in vivo molecular imaging biomarker for PD.

## Figures and Tables

**Table 1:** Main results of MRS studies in Parkinson's disease versus controls.

MRS technique	Number of PD patients	Main results versus control subjects	Mean (SD) of metabolite ratios in controls	Mean (SD) of metabolite ratios in PD patients	Significant differences were indicated by the following:	Reference
^ 1^HMRS at 1.5 T	6	Reduction of NAA/Cho ratios in the lentiform nucleus	NAA/Cho: 2.11 (0.39)	NAA/Cho: 1.29 (0.28)	*P* ≤ 0.02.	[9]

^ 1^HMRS at 1.5 T	15 (in 7 the symptomatic side was on the left and in 8 it was on the right)	Reduction of NAA/Cr ratios in the left symptomatic side of SN	NAA/Cr: 1.37 (0.22)	NAA/Cr: 1.20 (0.36)	*P* = 0.001	[10]

^ 1^HMRS at 1.5 T	10	Increase of total Cr in the prefrontal cortex	Cr: 0.61 (0.16) IU	Cr: 0.76 (0.08) IU	*P* < 0.05	[11]

^ 1^HMRS at 1.5 T	20	Reduction of NAA/Cr and Cho/Cr ratios in the temporoparietal cortex	NAA/Cr: 2.06 (∗) Cho/Cr: 0.75 (∗)	NAA/Cr: 1.78 (∗) Cho/Cr: 0.47 (∗)	For NAA/Cr *P* < 0.05 For Cho/Cr *P* < 0.01	[12]

^ 1^HMRS at 1.5 T	17	Reduction of NAA/Cr ratios in the temporoparietal cortex	NAA/Cr: 2.20 (0.38)	NAA/Cr: 1.78 (0.30)	*P* < 0.05	[13]

^ 1^HMRS at 1.5 T	10	Reduction of NAA/Cr ratios in the motor cortex	NAA/Cr: 1.34 (0.11)	NAA/Cr: 1.21 (0.12)	*P* < 0.05	[14]

^ 1^HMRS at 1.5 T	12	Reduction of NAA/Cr ratios in the posterior cingulated cortex	NAA/Cr: 1.78 (0.39)	NAA/Cr: 1.53 (0.20)	*P* = 0.03	[15]

^ 1^HMRS at 1.5 T	44	Reduction of NAA/Cr ratios in the presupplementary motor area	NAA/Cr: 1.47 (0.16)	NAA/Cr: 1.39 (0.17)	*P* = 0.045	[16]

3D ^1^HMRSI at 3 T	9	Reduction of NAA/Cr and mI/Cr in the rostral SN regions and increase of NAA/Cr and mI/Cr in the caudal SN regions	NAA/Cr_ros_: 3.34 (1.23) mI/Cr_ros_: 0.82 (0.43) NAA/Cr_cau_: 2.03 (0.67) mI/Cr_cau_: 0.64 (0.34)	NAA/Cr_ros_: 2.45 (1.55) mI/Cr_ros_: 0.59 (0.52) NAA/Cr_cau_: 4.92 (2.96) mI/Cr_cau_: 1.95 (1.43)	*P* = 0.054 for NAA/Cr_ros_ *P* = 0.248 for mI/Cr_ros_ *P* = 0.002 for NAA/Cr_cau_ *P* = 0.021 for mI/Cr_cau_	[21]

^ 1^HMRSI at 3 T	20	Reduction of NAA/Cr and Cho/Cr ratios in bilateral temporal gray matter and increase of total Cr in the right temporal gray matter	NAA/Cr_left_: 1.27 (0.09) Cho/Cr_left_: 0.18 (0.032) NAA/Cr_rigth_: 1.32 (0.121) Cho/Cr_left_: 0.18 (0.034) Cr_rigth_: 1995 (225) IU	NAA/Cr_left_: 1.13 (0.131) Cho/Cr_left_: 0.14 (0.028) NAA/Cr_rigth_: 1.17 (0.213) Cho/Cr_left_: 0.13 (0.034) Cr_rigth_: 2243 (361) IU	*P* ≤ 0.01 for NAA/Cr_left_ and Cho/Cr_left_ *P* ≤ 0.05 for NAA/Cr_right_ *P* ≤ 0.01 for Cho/Cr_right_ *P* ≤ 0.05 for Cr_right_	[22]

^ 1^HMRS at 3 T	12	Reduction of Glu/Cr ratios in the posterior cingulated gyrus	Glu/Cr: 0.555 (0.07)	Glu/Cr: 0.474 (0.092)	*P* = 0.019	[23]

^ 1^HMRS at 4 T	10	Reduction of Glu, NAA, and glutathione and increase of Cho in the SN. Increase of GABA/Glu ratio in SN versus cerebral cortex	NA	NA	NA	[24]

^ 1^HMRS at 7 T	13	Increase of GABA in the pons and putamen	GABA_pons_: 1.0 (0.2) *μ*mol/g GABA_putamen_: 1.6 (0.2) *μ*mol/g	GABA_pons_: 1.6 (0.4) *μ*mol/g GABA_putamen_: 2.1 (0.4) *μ*mol/g	*P* < 0.001 for GABA_pons_ *P* < 0.05 for GABA_putamen_	[25]

Cho: choline-containing compounds; Cr: creatine + phosphocreatine; GABA: *γ*-aminobutyric acid; Glu: glutamate; ^1^HMRS: proton magnetic resonance spectroscopy; ^1^HMRSI: proton magnetic resonance spectroscopy imaging; IU: institutional units; mI: myoinositol; MRS: magnetic resonance spectroscopy; NA: not applicable; NAA: N-acetylaspartate; PD: Parkinson's disease; SD: standard deviation; SN: substantia nigra; T: Tesla. ∗: DS not done.

**Table 2:** Main results of MRS studies in Parkinson's disease and atypical parkinsonian disorders.

MRS technique	Disease	Main results	Mean (SD) of metabolite ratios in controls	Mean (SD) of metabolite ratios in patients	Significant differences were indicated by the following:	Reference
^ 1^HMRS at 1.5 T	MSA-P and PD versus controls	Reduction of NAA/Cr ratios in the lentiform nucleus	NAA/Cr: 1.76 (0.96)	NAA/Cr_MSA-P_: 1.35 (0.29) NAA/Cr_PD_: 1.82 (0.28)	*P* < 0.02 MSA-P versus controls *P* > 0.05 PD versus controls	[17]

^ 1^HMRS at 1.5 T	PSP, CBD, MSA, and VP versus controls	Reduction of NAA/Cr ratios in the more affected frontal cortex	NAA/Cr: 2.1 (0.2)	NAA/Cr_PSP_: 1.4 (0.5) NAA/Cr_CBD_: 0.9 (0.4) NAA/Cr_MSA_: 1.2 (0.2) NAA/Cr_VP_: 1.7 (0.1)	*P* < 0.01 PSP, MSA and VP versus controls; *P* < 0.001 CBD versus controls	[37]
	PSP, MSA, CBD, and PD versus controls	Reduction of NAA/Cr ratios in the more affected putamen	NAA/Cr: 2.2 (0.2)	NAA/Cr_PSP_: 1.4 (0.2) NAA/Cr_CBD_: 1.0 (0.4) NAA/Cr_MSA_: 1.8 (0.3) NAA/Cr_PD_: 1.5 (0.2)	*P* < 0.01 PSP and PD versus controls; *P* < 0.001 CBD versus controls; *P* < 0.05 MSA versus controls	

		Reduction of NAA levels in the putamen	NAA: 7.1 (1.6) mM	NAA_PSP_: 5.26 (0.9) mM NAA_MSA-P_: 5.27 (0.6) mM NAA_PD_: 6.88 (1.2) mM	*P* = 0.003 MSA-P versus controls *P* = 0.023 MSA-P versus PD *P* = 0.002 PSP versus controls *P* = 0.016 PSP versus PD	
^ 1^HMRS at 1.5 T	MSA-P and PSP versus controls and PD	Reduction of NAA levels in the pallidum	NAA: 6.52 (1.5) mM	NAA_PSP_: 4.07 (1.0) mM NAA_MSA-P_: 5.54 (1.1) mM NAA_PD_: 6.36 (0.8) mM	*P* < 0.001 PSP versus controls *P* < 0.001 PSP versus PD	[38]
		Reduction of NAA levels in the lentiform nucleus	NAA: 6.77 (1.2) mM	NAA_PSP_: 4.6 (0.6) mM NAA_MSA-P_: 5.4 (0.7) mM NAA_PD_: 6.62 (0.8) mM	*P* = 0.003 MSA-P versus controls *P* = 0.027 MSA-P versus versus PD *P* < 0.001 PSP versus controls *P* < 0.001 PSP versus PD	

^ 1^HMRS at 1.5 T	MSA and PSP versus controls and PD	Reduction of NAA/Cho ratios in the lentiform nucleus	NAA/Cho: 2.02 (0.43)	NAA/Cho_MSA_: 1.39 (0.31) NAA/Cho_PSP_: 1.45 (0.28) NAA/Cho_PD_: 1.82 (0.28)	*P* < 0.001 MSA versus controls *P* < 0.001 PSP versus controls *P* < 0.001 MSA versus PD *P* < 0.05 PSP versus PD	[39]
^ 1^HMRS at 1.5 T	MSA, PSP, and PD versus controls and MSA versus PD	Reduction of NAA/Cr ratios in the lentiform nucleus	NAA/Cr: 1.86 (0.29)	NAA/Cr_MSA_: 1.32 (0.30) NAA/Cr_PSP_: 1.40 (0.17) NAA/Cr_PD_: 1.65 (0.41)	*P* < 0.001 MSA versus controls *P* < 0.001 PSP versus controls *P* < 0.05 PD versus controls *P* < 0.01 MSA versus PD	

^ 1^HMRS at 3 T	MSA-C and MSA-P versus controls; MSA-P versus PD	Reduction of NAA/Cr ratios in the pontine basis	NA	NA	*P* < 0.0001 MSA-C versus controls *P* < 0.0001 MSA-P versus controls *P* = 0.001 MSA-P versus PD	[40]
	MSA-P versus controls and PD	Reduction of NAA/Cr ratios in the putamen	NA	NA	*P* = 0.009 MSA-P versus controls *P* = 0.002 MSA-P versus PD	

3D ^1^HMRSI at 3 T	PD versus controls and ADPs	Lower NAA/Cr ratios in the rostral SN regions than those in the caudal SN regions	(NAA/Cr)_ros_: 2.56 (0.73) and (NAA/Cr)_cau_: 1.85 (0.51)	In PD (NAA/Cr)_ros_: 1.97 (1.24) and (NAA/Cr)_cau_: 3.47 (2.37). In ADPs (NAA/Cr)_ros_: 2.29 (1.37) and (NAA/Cr)_cau_: 2.09 (0.92)	*P* < 0.0001 PD versus controls; *P* < 0.0001 PD versus APDs	[41]
	APDs versus controls	Lower NAA/Cr ratios in the caudal SN regions than those in the rostral SN regions	(NAA/Cr)_ros_: 2.56 (0.73) and (NAA/Cr)_cau_: 1.85 (0.51)	(NAA/Cr)_ros_: 2.29 (1.37) and (NAA/Cr)_cau_: 2.09 (0.92)	*P* = 0.977 APDs versus controls	

APDs: atypical parkinsonian disorders; CBD: corticobasal degeneration; Cho: choline-containing compounds; Cr: creatine + phosphocreatine; ^1^HMRS: proton magnetic resonance spectroscopy; ^1^HMRSI: proton magnetic resonance spectroscopy imaging; MRS: magnetic resonance spectroscopy; MSA: multiple-system atrophy; MSA-C: cerebellar ataxia variant of multiple-system atrophy; MSA-P: Parkinson's variant of multiple-system atrophy; NA: not applicable; NAA: N-acetylaspartate; PD: Parkinson's disease; PSP: progressive supranuclear palsy; SD: standard deviation; SN: substantia nigra; T: Tesla; VP: vascular parkinsonism.

**Table 3:** Main results of MRS studies in PD patients treated with dopaminergic drugs and DBS.

MRS technique	Drug	Main results	Mean (SD) of metabolite ratios in treated patients	Mean (SD) of metabolite ratios in nontreated patients	Mean (SD) of metabolite ratios in controls	Significant differences were indicated by the following:	Reference
^ 1^HMRS at 1.5 T	Levodopa/carbidopa	Low NAA/Cho ratios in striatum of nontreated PD patients	NAA/Cho: 1.80 (0.48)	NAA/Cho: 1.60 (0.33)	NAA/Cho: 1.83 (0.62)	*P* = 0.012 for nontreated patients versus controls	[45]
^ 1^HMRS at 1.5 T	Levodopa	Reduction in putaminal NAA/Cho ratios contralateral to the most affected side in drug-naïve patients	NAA/Cho: 1.15 (0.19)	NAA/Cho: 0.97 (0.14)	NAA/Cho: 1.26 (0.28)	*P* = 0.009 for nontreated patients versus controls	[46]

MRS technique	Treatment	Main results	Mean (SD) of metabolite ratios before treatment	Mean (SD) of metabolite ratios after treatment	Mean (SD) of metabolite ratios in controls	Significant differences were indicated by the following:	Reference

^ 1^HMRS at 1.5 T	Pergolide	Significant increase of Cho/Cr ratios in the motor cortex after therapy	Cho/Cr: 0.71 (0.13)	Cho/Cr: 0.82 (0.13)	NA	*P* < 0.05	[47]
^ 1^HMRS at 1.0 T	DBS of the STN	Cortical increase of NAA/Cho and NAA/Cr ratios	NAA/Cho: 1.3238 (0.3196) NAA/Cr: 1.6303 (0.6361)	NAA/Cho: 2.5583 (1.2993) NAA/Cr: 1.7057 (0.4167)	NA	*P* = 0.04699 for NAA/Cho; *P* = 0.0326 for NAA/Cr	[48]

Cho: choline-containing compounds; Cr: creatine + phosphocreatine; DBS: deep brain stimulation; ^1^HMRS: proton magnetic resonance spectroscopy; MRS: magnetic resonance spectroscopy; NA: not applicable; NAA: N-acetylaspartate; PD: Parkinson's disease; SD: standard deviation; STN: subthalamic nucleus; T: Tesla.
